# Dietary metal intake and the prevalence of erectile dysfunction in US men: Results from National Health and Nutrition Examination Survey 2001–2004

**DOI:** 10.3389/fnut.2022.974443

**Published:** 2022-11-03

**Authors:** Rui-Ji Liu, Shu-Ying Li, Zhi-Peng Xu, Jun-Jie Yu, Wei-Pu Mao, Chao Sun, Bin Xu, Ming Chen

**Affiliations:** ^1^Department of Urology, People’s Hospital of Putuo District, Shanghai, China; ^2^Department of Urology, Affiliated Zhongda Hospital of Southeast University, Nanjing, China; ^3^Surgical Research Center, Institute of Urology, Southeast University Medical School, Nanjing, China; ^4^Sichuan Cancer Hospital and Institute, Sichuan Cancer Center, Cancer Hospital Affiliate to School of Medicine, University of Electronic Science and Technology of China, Chengdu, China; ^5^Nanjing Lishui District People’s Hospital, Zhongda Hospital Lishui Branch, Southeast University, Nanjing, China

**Keywords:** dietary trace metals intake, erectile dysfunction, nutrition, atherosclerosis, National Health and Nutrition Examination Survey (NHANES)

## Abstract

**Background:**

Erectile dysfunction (ED) mainly affects men over 40 years of age and is a common clinical condition. In addition to hypertension and diabetes, environment, and lifestyle are also significantly associated with erectile dysfunction. The relationship between dietary trace metal intake and ED has not been studied.

**Materials and methods:**

Data on participants were obtained from the National Health and Nutrition Examination Survey for this study, and those with incomplete information on clinical variables were excluded. Dose-response curve analysis was used to investigate the relationship between dietary trace metal intake and ED prevalence. Multivariate logistic regression analysis was used to adjust for confounders to further investigate the relationship between dietary trace metal intake and ED prevalence. 1:1 propensity score matching (PSM) was performed to adjust for differences between clinical variables for data reanalysis to confirm the reliability of the results.

**Results:**

A total of 3,745 individuals were included in the study, including 1096 ED patients and 2,649 participants without ED. Dietary intake of trace metals (Mg, Zn, Cu, and Se) was significantly higher in participants without ED than in ED patients (all *P* < 0.001). Dose-response curve analysis showed a significant negative association between these dietary metal intakes and ED prevalence (all *P* < 0.001). Multivariate logistic regression analysis adjusted for confounders (age, education, BMI, annual household income, hypertension, diabetes, marital status, race, and current health status) revealed that increased dietary metal intake reduced the odds ratio of ED. 1:1 PSM reanalysis further confirmed the validity of the results.

**Conclusion:**

Increasing dietary intake of trace metals (magnesium, zinc, copper, and selenium) within the upper limit is beneficial in reducing the prevalence of ED.

## Introduction

Erectile dysfunction (ED) is a common clinical condition that commonly affects men over the age of 40. The National Institutes of Health (NIH) defines erectile dysfunction as the inability to maintain a sufficient penile erection over the course of a sexual relationship (1). Incidence of ED gradually increases with age. International Consultation Committee for Sexual Medicine on Definitions/Epidemiology/Risk Factors for Sexual Dysfunction states that the prevalence of ED in men less than 40 years of age is 1–10%. However, it increases to 20–40% in men aged 60–69 years, and ranges from 50 to 100% in those over 70 years (2–4). Another study showed that ED prevalence among men aged 60–69 years reached 46 per 1,000 person-years (5). As the world’s population ages, the prevalence of ED will be even higher, with 322 million cases expected by 2025 (6, 7).

Several studies have shown that ED is closely related to hypertension, diabetes, hyperlipidemia, and neurological disorders (8–10). Both vascular and neurological related lesions can lead to ED. Penile arteries are typically 1 to 2 mm in diameter, smaller than the 3 to 4 mm of coronary arteries and the 5 to 7 mm of carotid arteries. Therefore, atherosclerotic plaques of a smaller size should be more likely to obstruct and affect the hemodynamics of the penile arteries. In addition, vascular endothelial dysfunction may also be involved in ED (11). Some studies have also shown that certain environments and lifestyles are also important predictors of ED, such as smoking, caffeine intake, Vitamin D deficiency, lack of physical activity, etc., (12–15). Lifestyle and nutritional intake changes may improve ED (16–18).

Electrolyte balance in the human body is a prerequisite for normal physiological activity. Essential trace metals intake is beneficial to health. Trace metals are components of some important metalloenzymes, involved in antioxidant and cholesterol metabolism. Deficiency of trace metals can lead to stroke, atherosclerosis, thrombosis, hypertension, and other conditions (19). However, there is a lack of studies on the relationship of dietary metal intake and ED prevalence.

## Materials and methods

### Data sources and population

The study population was obtained from the National Health and Nutrition Examination Survey (NHANES), a program of studies designed to assess the health and nutritional status of non-institutionalized civilians in the United States.^1^ The survey combines interviews and physical examinations involving a variety of health and nutrition measures. A nationally representative sample of approximately 5,000 individuals is surveyed each year, and interviews include demographic, socioeconomic, dietary, and health-related questions. The examination component includes medical, dental and physiological measurements conducted by trained medical personnel.

We obtained data of NHANES participants (men ≥ 20 years of age) between 2001 and 2004. Lack of relevant data was excluded: incomplete data on erectile dysfunction, incomplete data on dietary metal intake; unknown body mass index (BMI), unknown marital status, unknown education level, unknown annual household income, unknown current health status and hypertension/diabetes.

### Study variables

Dietary metal intake data of interest (magnesium, zinc, copper, and selenium) were obtained for this study. The types and amounts of food and beverages consumed in the 24 h prior to the interview (midnight to midnight) were obtained from NHANES participants through dietary interviews. Then, the energy, nutrient, and other food components consumed from these foods and beverages were estimated by the USDA’s Food and Nutrient Database for Dietary Studies, 2.0 (FNDDS 2.0).

We also obtained other variables known or potentially associated with ED, such as age (less or more than 40 years), education level (less or more than a high school diploma), race (Mexican American, other Hispanic, non-Hispanic white, non-Hispanic black, other race), BMI (less or more than 25 kg/m^2^) annual household income (less or more than $20,000), marital status (yes/no), hypertension (yes/no), diabetes (yes, no, borderline), current health status (excellent, very good, good; fair or poor).

### Erectile dysfunction assessment

According to Massachusetts Male Aging Study (MMAS) (20), trained interviewers asked male participants 20 years and older a single question to assess their ability to maintain an erection: “Many men experience problems with sexual intercourse. How would you describe your ability to get and keep an erection adequate for satisfactory intercourse?” Participants had the following options to describe their erectile response: “always able or almost always able to get and keep an erection,” “usually able to get and keep an erection,” “sometimes able to get and keep an erection,” “never able to get and keep an erection.” We define “sometimes able or never able” as having erectile dysfunction and “always or almost always able and usually able” as not having erectile dysfunction, which is consistent with previous studies (12, 13, 21, 22).

### Statistical analysis

All statistical analyses were performed using R software (version 4.2.0) and SPSS (version 26). For categorical variables, data were described by frequency (n) and percentage (%). *P* values were analyzed by chi-square tests. For non-normally distributed continuous variables, data were described by interquartile range (IQR). *P* values were analyzed by Wilcoxon rank sum test. Multivariate logistic regression models were used to assess the relationship between different dietary metal intakes and ED, resulting in adjusted odds ratios (OR) and 95% confidence intervals (CI). Dose-response relationship between dietary metal intake and ED were described by restricted cubic spline (RCS) function. Subgroup analysis was also performed according to age, marital status and BMI.

Differences between ED and non-ED were balanced by 1:1 propensity score matching (PSM). Baseline characteristics were adjusting for confounding variables including: age, race, marital status, education level, BMI, hypertension, diabetes, annual household income, and current health status. The obtained PSM data were also re-analyzed to further test the correctness of the results. *P* value < 0.05 was considered statistically significant.

## Results

**FIGURE 1:**
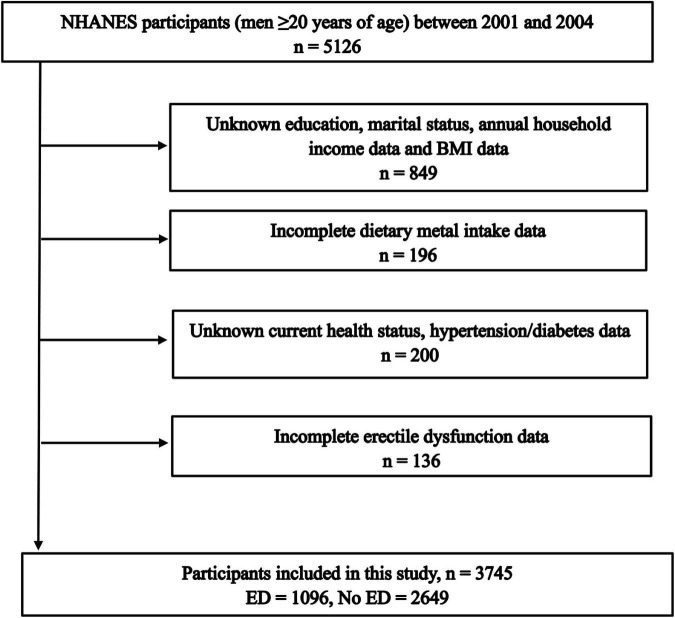
Flow chart of the study population identification.

Excluding participants with incomplete information, a total of 3,745 participants were included in the study, including 1096 (29.27%) ED patients and 2649 (70.73%) participants without ED (Figure 1). The number of people younger than 40 years of age with ED was only 73 (1.9%), and the percentage of people older than 40 years of age with ED was 27.3% (1023). In addition, participants with higher levels of education were less likely to have ED. Similarly, high income, health status, absence of hypertension and diabetes, and low BMI was associated with decreased ED incidence (Table 1). Interestingly, dietary magnesium, zinc, copper, and selenium intakes were significantly lower in ED patients than participants without ED (all *P* < 0.001).

**TABLE 1:** Baseline characteristics of national health and nutrition examination survey (NHANES) participants between 2001 and 2004 before propensity score matching (PSM).

Characteristic	Non-ED	ED	*p*
Total patients	2,649	1,096	
Age (years), n (%)			< 0.001
< 40 years	1130 (30.2%)	73 (1.9%)	
≥ 40 years	1519 (40.6%)	1023 (27.3%)	
Race, n (%)			< 0.001
Mexican American	512 (13.7%)	202 (5.4%)	
Other hispanic	85 (2.3%)	38 (1%)	
Non-hispanic white	1428 (38.1%)	674 (18%)	
Non-hispanic black	531 (14.2%)	160 (4.3%)	
Other race–including multi-racial	93 (2.5%)	22 (0.6%)	
Education level, n (%)			< 0.001
Less than high school	592 (15.8%)	431 (11.5%)	
High school diploma	692 (18.5%)	220 (5.9%)	
More than high school	1365 (36.4%)	445 (11.9%)	
Marital status, n (%)			< 0.001
Married	1580 (42.2%)	784 (20.9%)	
Unmarried	1069 (28.5%)	312 (8.3%)	
Annual household income, n (%)			< 0.001
Over $20,000	2190 (58.5%)	788 (21%)	
Under $20,000	459 (12.3%)	308 (8.2%)	
BMI (kg/m^2^), n (%)			0.010
< 25.0	805 (21.5%)	286 (7.6%)	
≥ 25.0	1844 (49.2%)	810 (21.6%)	
Hypertension, n (%)			< 0.001
Yes	631 (16.8%)	584 (15.6%)	
No	2018 (53.9%)	512 (13.7%)	
Current health status, n (%)			< 0.001
Excellent, very good, good	2264 (60.5%)	733 (19.6%)	
Fair or poor	385 (10.3%)	363 (9.7%)	
Diabetes, n (%)			< 0.001
Yes	134 (3.6%)	256 (6.8%)	
No	2485 (66.4%)	822 (21.9%)	
Borderline	30 (0.8%)	18 (0.5%)	
Magnesium (mg), median (IQR)	301 (217, 407)	256.5 (185.75, 335)	< 0.001
Zinc (mg), median (IQR)	12.76 (8.49, 18.04)	10.1 (6.93, 14.64)	< 0.001
Copper (mg), median (IQR)	1.34 (0.94, 1.83)	1.1 (0.82, 1.5)	< 0.001
Selenium (mcg), median (IQR)	117.3 (85.1, 160.7)	97.4 (71.57, 130.83)	< 0.001

For categorical variables, *P* values were analyzed by chi-square tests. For continuous variables, *P* values were analyzed by Wilcoxon rank sum test. BMI, body mass index; PSM, propensity score matching; IQR, interquartile range.

**FIGURE 2:**
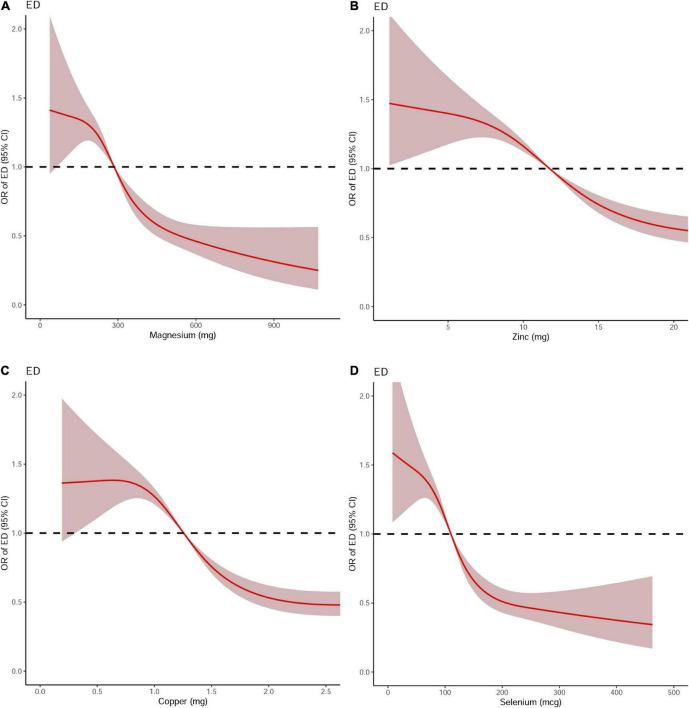
The dose-response analysis of dietary metal intake and erectile dysfunction before propensity score matching. **(A)** Magnesium; **(B)** Zinc; **(C)** Copper; and **(D)** Selenium.

**FIGURE 3:**
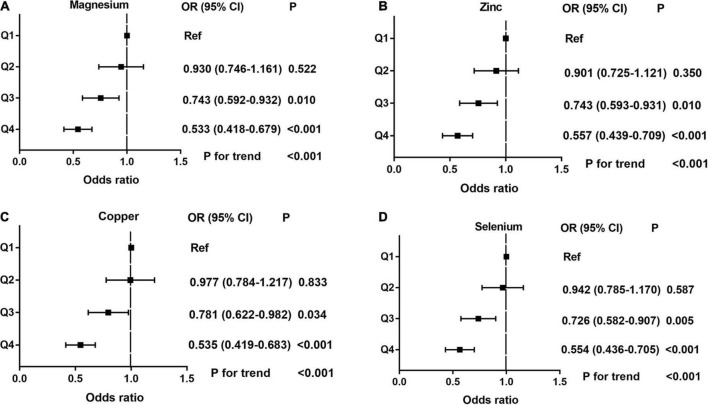
The adjusted odds ratios (95% CI) of dietary metal intake and erectile dysfunction before propensity score matching. **(A)** Magnesium; **(B)** Zinc; **(C)** Copper; and **(D)** Selenium.

Dose-response curve analysis of the RCS showed that dietary magnesium, zinc, copper, and selenium intake were negatively and non-linearly correlated with ED (all *P* < 0.001). The prevalence of ED decreased with increasing dietary trace metal intake (Figure 2). In addition, we performed subgroup analyses according to age, marital status, and BMI and obtained the same results, except in participants younger than 40 years. Interestingly, dietary magnesium, copper, and selenium (except zinc) intakes were not significantly associated with ED incidence in participants younger than 40 years of age but were significantly associated in participants older than 40 years of age (Table 2). Multivariate logistic regression models analysis adjusted for confounders (age, race, marital status, annual household income, education level, BMI, current health status, hypertension, diabetes) showed that the OR for ED decreased significantly with increased dietary intake of trace metals (Q1–Q4) (all *P* < 0.001) (Figure 3 and Table 3).

**TABLE 2:** Subgroup analysis of dietary metal intake and erectile dysfunction in national health and nutrition examination survey (NHANES) 2001–2004 before propensity score matching (PSM).

Characteristic	OR (95% CI)			*P* value
**Magnesium**	**100 mg/day**	**200 mg/day**	**300 mg/day**	

Overall	1.375 (1.069–1.769)	1.285 (1.185–1.393)	0.933 (0.909–0.957)	< 0.0001
< 40 years	1.488 (0.677–3.270)	1.311 (0.982–1.750)	1.028 (0.990–1.068)	0.1747
≥ 40 years	1.356 (1.013–1.816)	1.233 (1.133–1.343)	0.923 (0.887–0.961)	< 0.0001
BMI < 25.0 kg/m^2^	1.528 (0.998–2.340)	1.426 (1.209–1.681)	0.959 (0.938–0.981)	< 0.0001
BMI ≥ 25.0 kg/m^2^	1.356 (0.991–1.856)	1.243 (1.133–1.363)	0.938 (0.907–0.970)	< 0.0001
Married	1.525 (1.110–2.096)	1.331 (1.202–1.475)	0.952 (0.931–0.974)	< 0.0001
Unmarried	1.333 (0.891–1.994)	1.271 (1.121–1.441)	0.906 (0.845–0.960)	< 0.0001

**Zinc**	**10 mg/day**	**20 mg/day**	**30 mg/day**	

Overall	1.152 (1.115–1.189)	0.564 (0.479–0.664)	0.531 (0.438–0.644)	< 0.0001
< 40 years	1.227 (1.020–1.475)	0.909 (0.595–1.387)	1.045 (0.597–1.825)	0.0475
≥ 40 years	1.075 (1.049–1.102)	0.595 (0.491–0.719)	0.563 (0.452–0.701)	< 0.0001
BMI < 25.0 kg/m^2^	1.375 (1.265–1.493)	0.334 (0.242–0.462)	0.329 (0.223–0.484)	< 0.0001
BMI ≥ 25.0 kg/m^2^	1.116 (1.072–1.162)	0.690 (0.571–0.834)	0.642 (0.521–0.804)	< 0.0001
Married	1.159 (1.114–1.206)	0.558 (0.458–0.681)	0.536 (0.423–0.677)	< 0.0001
Unmarried	1.161 (0.084–1.242)	0.613 (0.459–0.817)	0.548 (0.384–0.781)	< 0.0001

**Copper**	**1 mg/day**	**2 mg/day**	**3 mg/day**	

Overall	1.250 (1.197–1.305)	0.534 (0.458–0.622)	0.482 (0.402–0.579)	< 0.0001
< 40 years	1.201 (0.986–1.464)	0.864 (0.558–1.340)	0.813 (0.445–1.485)	0.3127
≥ 40 years	1.175 (1.128–1.225)	0.563 (0.470–0.674)	0.539 (0.440–0.660)	< 0.0001
BMI < 25.0 kg/m^2^	1.342 (1.225–1.471)	0.451 (0.333–0.610)	0.338 (0.228–0.502)	< 0.0001
BMI ≥ 25.0 kg/m^2^	1.244 (1.176–1.316)	0.566 (0.472–0.680)	0.553 (0.447–0.684)	< 0.0001
Married	1.296 (1.216–1.381)	0.569 (0.472–0.686)	0.534 (0.430–0.664)	< 0.0001
Unmarried	1.241 (1.162–1.324)	0.455 (0.338–0.613)	0.403 (0.285–0.572)	< 0.0001

**Selenium**	**100 mcg/day**	**200 mcg/day**	**300 mcg/day**	

Overall	1.121 (1.086–1.158)	0.510 (0.430–0.606)	0.431 (0.317–0.585)	< 0.0001
< 40 years	1.093 (0.910–1.314)	1.050 (0.634–0.1740)	1.049 (0.487–2.257)	0.1210
≥ 40 years	1.056 (1.034–1.077)	0.548 (0.452–0.664)	0.478 (0.342–0.668)	< 0.0001
BMI < 25.0 kg/m^2^	1.147 (1.086–1.211)	0.340 (0.240–0.481)	0.258 (0.129–0.513)	< 0.0001
BMI ≥ 25.0 kg/m^2^	1.095 (1.057–1.134)	0.591 (0.485–0.720)	0.514 (0.362–0.731)	< 0.0001
Married	1.118 (1.075–1.163)	0.553 (0.451–0.678)	0.515 (0.362–0.733)	< 0.0001
Unmarried	1.135 (1.072–1.200)	0.452 (0.329–0.620)	0.339 (0.189–0.611)	< 0.0001

CI, confidence interval; OR, odds ratio; PSM, propensity score matching.

**TABLE 3:** Multivariate logistic regression analysis of the relationship between dietary metal intake and erectile dysfunction in national health and nutrition examination survey (NHANES) 2001–2004 before propensity score matching (PSM).

Metals	Magnesium		Zinc		Copper		Selenium	
				
	OR* (95% CI)	*P*	OR* (95% CI)	*P*	OR* (95% CI)	*P*	OR* (95% CI)	*P*
Q1	Ref		Ref		Ref		Ref	
Q2	0.930 (0.746–1.161)	0.522	0.901 (0.725–1.121)	0.350	0.977 (0.784–1.217)	0.833	0.942 (0.785–1.170)	0.587
Q3	0.743 (0.592–0.932)	0.010	0.743 (0.593–0.931)	0.010	0.781 (0.622–0.982)	0.034	0.726 (0.582–0.907)	0.005
Q4	0.533 (0.418–0.679)	< 0.001	0.557 (0.439–0.709)	< 0.001	0.535 (0.419–0.683)	< 0.001	0.554 (0.436–0.705)	< 0.001
P for trend	< 0.001		< 0.001		< 0.001		< 0.001	

CI, confidence interval; OR, odds ratio; PSM, propensity score matching; *adjust for age, race, education level, marital status, annual household income, BMI, hypertension, diabetes, current health status.

**FIGURE 4:**
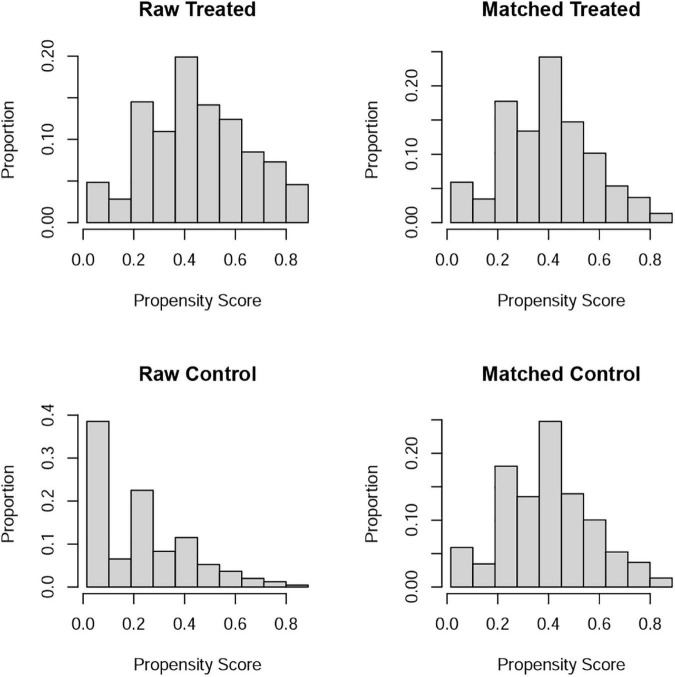
Propensity score matching analysis for treated and control population.

**FIGURE 5:**
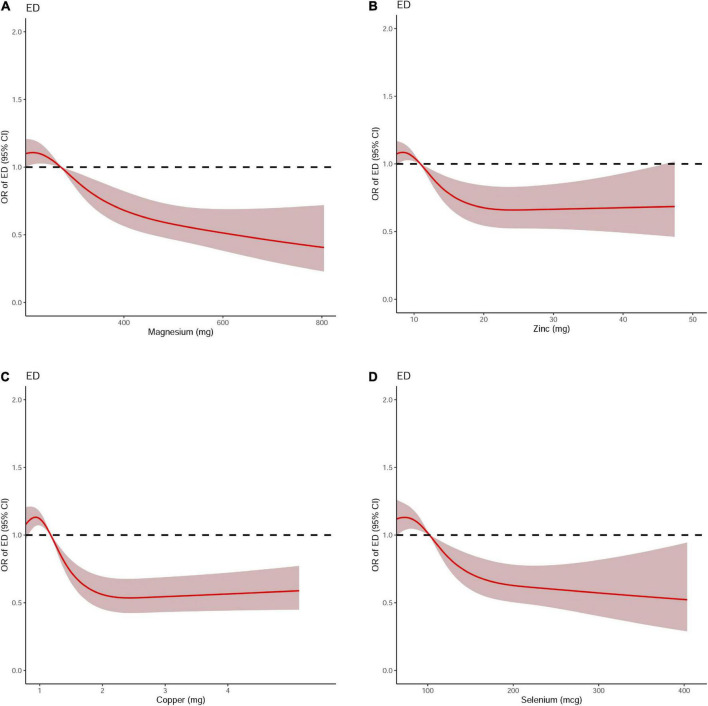
The dose-response analysis of dietary metal intake and erectile dysfunction after propensity score matching. **(A)** Magnesium; **(B)** Zinc; **(C)** Copper; and **(D)** Selenium.

**FIGURE 6:**
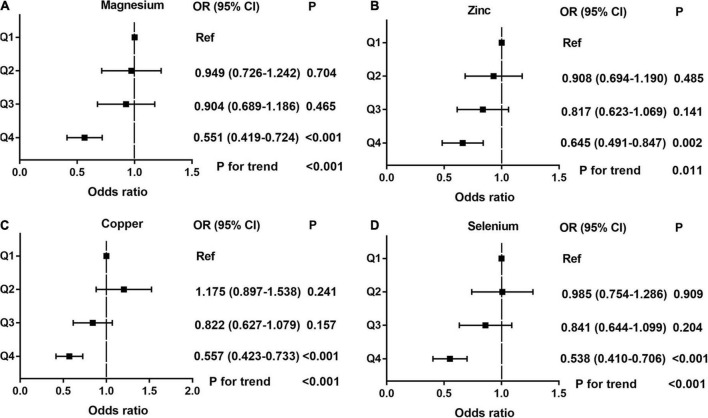
The adjusted odds ratios (95% CI) of dietary metal intake and erectile dysfunction after propensity score matching. **(A)** Magnesium; **(B)** Zinc; **(C)** Copper; and **(D)** Selenium.

Differences (age, race, marital status, education level, BMI, hypertension, diabetes, annual household income, and current health status) between ED and non-ED were balanced by 1:1 PSM (Figure 4). After PSM, there were 896 ED patients and 896 participants without ED. Dietary metal (magnesium, zinc, copper, selenium) intake remained significantly higher in participants without ED than in ED patients (Table 4). After PSM reanalysis, dose-response curve analysis of RCS again verified that dietary intake of magnesium, zinc, copper, and selenium was negatively and non-linearly related to ED (Figure 5). Subgroup analyses by age, marital status, and BMI also showed a significant negative association between dietary trace metal intake and ED prevalence (except in participants younger than 40 years) (Table 5). After PSM, multivariate logistic regression analysis also resulted in a decrease in ED prevalence with increasing dietary trace metal intake (Figure 6 and Table 6). Food sources of trace metals and recommended upper limit were summarized and displayed in Table 7.

**TABLE 4:** Baseline characteristics of national health and nutrition examination survey (NHANES) participants between 2001 and 2004 after propensity score matching (PSM).

Characteristic	Non-ED	ED	*p*
Total patients	896	896	
Age (years), n (%)			0.864
< 40 years	76 (4.2%)	73 (4.1%)	
≥ 40 years	820 (45.8%)	823 (45.9%)	
Race, n (%)			0.005
Mexican American	160 (8.9%)	155 (8.6%)	
Other hispanic	26 (1.5%)	32 (1.8%)	
Non-hispanic white	529 (29.5%)	584 (32.6%)	
Non-hispanic black	161 (9%)	106 (5.9%)	
Other race–including multi-racial	20 (1.1%)	19 (1.1%)	
Education level, n (%)			0.171
Less than high school	302 (16.9%)	315 (17.6%)	
High school diploma	215 (12%)	182 (10.2%)	
More than high school	379 (21.1%)	399 (22.3%)	
Marital status, n (%)			1.000
Married	625 (34.9%)	624 (34.8%)	
Unmarried	271 (15.1%)	272 (15.2%)	
Annual household income, n (%)			0.482
Over $20,000	659 (36.8%)	673 (37.6%)	
Under $20,000	237 (13.2%)	223 (12.4%)	
BMI (kg/m^2^), n (%)			1.000
< 25.0	236 (13.2%)	237 (13.2%)	
≥ 25.0	660 (36.8%)	659 (36.8%)	
Hypertension, n (%)			0.297
Yes	428 (23.9%)	405 (22.6%)	
No	468 (26.1%)	491 (27.4%)	
Current health status, n (%)			0.418
Excellent, very good, good	673 (37.6%)	657 (36.7%)	
Fair or poor	223 (12.4%)	239 (13.3%)	
Diabetes, n (%)			0.017
Yes	115 (6.4%)	140 (7.8%)	
No	774 (43.2%)	738 (41.2%)	
Borderline	7 (0.4%)	18 (1%)	
Magnesium (mg), median (IQR)	280 (202, 393.25)	260 (191, 338.25)	< 0.001
Zinc (mg), median (IQR)	11.41 (7.55, 16.5)	10.34 (7.06, 14.74)	0.002
Copper (mg), median (IQR)	1.25 (0.88, 1.72)	1.11 (0.84, 1.5)	< 0.001
Selenium (mcg), median (IQR)	108 (78.15, 147.25)	98.4 (71.68, 130.75)	< 0.001

For categorical variables, *P* values were analyzed by chi-square tests. For continuous variables, *P* values were analyzed by Wilcoxon rank sum test. BMI, body mass index; PSM, propensity score matching. IQR, interquartile range.

**TABLE 5:** Subgroup analysis of dietary metal intake and erectile dysfunction in national health and nutrition examination survey (NHANES) 2001–2004 after propensity score matching (PSM).

Characteristic	OR (95% CI)			*P* value
**Magnesium**	**100 mg/day**	**200 mg/day**	**300 mg/day**	

Overall	1.012 (1.005–1.019)	0.626 (0.503–0.780)	0.572 (0.402–0.815)	< 0.0001
< 40 years	1.311 (0.494–3.477)	1.244 (0.897–1.726)	0.911 (0.780–1.064)	0.547
≥ 40 years	0.811 (0.571–1.153)	1.077 (0.980–1.182)	0.906 (0.845–0.970)	< 0.0001
BMI < 25.0 kg/m^2^	0.728 (0.402–1.319)	1.093 (0.919–1.301)	0.867 (0.755–0.996)	0.0011
BMI ≥ 25.0 kg/m^2^	0.887 (0.583–1.347)	1.100 (0.981–1.234)	0.913 (0.848–0.983)	0.0096
Married	0.755 (0.488–1.167)	1.052 (0.933–1.186)	0.929 (0.870–0.993)	0.0020
Unmarried	0.976 (0.563–1.692)	1.151 (1.010–1.313)	0.854 (0.754–0.968)	0.0059

**Zinc**	**10 mg/day**	**20 mg/day**	**30 mg/day**	

Overall	1.048 (1.019–1.077)	0.677 (0.545–0.841)	0.665 (0.520–0.850)	0.0039
< 40 years	0.997 (0.803–1.239)	1.098 (0.617–1.954)	1.185 (0.602–2.330)	0.902
≥ 40 years	1.044 (1.019–1.071)	0.634 (0.503–0.799)	0.606 (0.464–0.791)	0.0010
BMI < 25.0 kg/m^2^	1.035 (1.016–1.054)	0.415 (0.263–0.655)	0.419 (0.255–0.689)	0.0021
BMI ≥ 25.0 kg/m^2^	1.031 (0.996–1.068)	0.779 (0.606–1.001)	0.755 (0.566–1.007)	0.231
Married	1.044 (1.012–1.078)	0.672 (0.516–0.874)	0.662 (0.493–0.888)	0.0295
Unmarried	1.036 (0.996–1.077)	0.669 (0.451–0.992)	0.645 (0.397–1.047)	0.1746

**Copper**	**1 mg/day**	**2 mg/day**	**3 mg/day**	

Overall	1.119 (1.070–1.170)	0.559 (0.451–0.692)	0.544 (0.430–0.689)	< 0.0001
< 40 years	1.130 (0.880–1.451)	0.842 (0.438–1.618)	0.945 (0.442–2.022)	0.786
≥ 40 years	1.123 (1.073–1.175)	0.536 (0.429–0.671)	0.523 (0.410–0.668)	< 0.0001
BMI < 25.0 kg/m^2^	1.081 (1.017–1.149)	0.500 (0.322–0.774)	0.422 (0.252–0.704)	0.0095
BMI ≥ 25.0 kg/m^2^	1.134 (1.069–1.204)	0.557 (0.432–0.719)	0.656 (0.468–0.918)	0.0001
Married	1.149 (1.070–1.235)	0.543 (0.419–0.703)	0.586 (0.429–0.795)	0.0001
Unmarried	1.100 (0.036–1.168)	0.549 (0.373–0.802)	0.529 (0.349–0.802)	0.0180

**Selenium**	**100 mcg/day**	**200 mcg/day**	**300 mcg/day**	

Overall	1.012 (1.005–1.019)	0.626 (0.503–0.780)	0.572 (0.402–0.815)	0.0001
< 40 years	0.975 (0.774–1.227)	1.230 (0.643–2.350)	1.350 (0.590–3.093)	0.885
≥ 40 years	1.016 (1.007–1.025)	0.578 (0.459–0.726)	0.512 (0.348–0.752)	< 0.0001
BMI < 25.0 kg/m^2^	1.003 (1.000–1.005)	0.430 (0.280–0.662)	0.378 (0.215–0.664)	0.0019
BMI ≥ 25.0 kg/m^2^	1.027 (1.004–1.051)	0.782 (0.566–0.936)	0.703 (0.459–1.077)	0.0102
Married	1.025 (1.008–1.041)	0.611 (0.471–0.794)	0.586 (0.384–0.893)	0.0031
Unmarried	0.998 (0.996–1.000)	0.659 (0.446–0.975)	0.570 (0.325–1.001)	0.025

CI, confidence interval; OR, odds ratio; PSM, propensity score matching.

**TABLE 6:** Multivariate logistic regression analysis of the relationship between dietary metal intake and erectile dysfunction in national health and nutrition examination survey (NHANES) 2001–2004 after propensity score matching (PSM).

Metals	Magnesium		Zinc		Copper		Selenium	
				
	OR* (95% CI)	*P*	OR* (95% CI)	*P*	OR* (95% CI)	*P*	OR* (95% CI)	*P*
Q1	Ref		Ref		Ref		Ref	
Q2	0.949 (0.726–1.242)	0.704	0.908 (0.694–1.190)	0.485	1.175 (0.897–1.538)	0.241	0.985 (0.754–1.286)	0.909
Q3	0.904 (0.689–1.186)	0.465	0.817 (0.623–1.069)	0.141	0.822 (0.627–1.079)	0.157	0.841 (0.644–1.099)	0.204
Q4	0.551 (0.419–0.724)	< 0.001	0.645 (0.491–0.847)	0.002	0.557 (0.423–0.733)	< 0.001	0.538 (0.410–0.706)	< 0.001
P for trend	< 0.001		0.011		< 0.001		< 0.001	

CI, confidence interval; OR, odds ratio; PSM, propensity score matching; *adjust for age, race, education level, marital status, annual household income, BMI, hypertension, diabetes, current health status.

**TABLE 7:** Food sources of trace metals and recommended upper limit.

Metals	Food sources of trace metals	Dietary reference upper limit
Magnesium	Grains, green leafy vegetables, almonds, coffee, and dark chocolate	420 mg/day
Zinc	Dry fruits (especially pine nuts, peanuts, nuts and almonds), all kind of meat, milk, cereals, and eggs	40 mg/day
Copper	Dry fruits, pine nuts, hazelnuts, soy beans, black-eyed beans/peas and lentils, dark chocolate	10 mg/day
Selenium	Fish, seafood, eggs, offal, and red meat	400 mcg/day

## Discussion

Erectile dysfunction primarily affects men over the age of 40. Typical causes of ED are diabetes and hypertension. In addition, some common lifestyle factors such as obesity, lack of physical activity, and alcohol consumption are also reported to be associated with the development of ED. A strong association between ED and cardiovascular disease has been demonstrated, and it was indicated to be a promising predictor of coronary artery disease (8).

Currently, the number of studies on the relationship between dietary nutritional intake and ED prevalence are limited. The aim of this study was to investigate the relationship between dietary trace metal intake and erectile dysfunction. The results showed that dietary intake of magnesium, zinc, copper, and selenium was significantly lower in ED patients than participants without ED. Dose-response curve analysis of the RCS also found a negative correlation between dietary trace metal intake (magnesium, zinc, copper, and selenium) and ED prevalence. Interestingly, in the subgroup analysis, dietary metal intake was not significantly associated with ED prevalence in participants younger than 40 years. This suggests that dietary trace metal intake primarily affects ED prevalence in men over 40 years of age. Multivariate logistic regression model analysis adjusted for confounders also showed that increasing dietary metal intake significantly reduced the OR of ED. The 1:1 PSM reanalysis further confirmed the correctness of the above results.

Foods such as grains, green leafy vegetables, almonds, coffee and dark chocolate contain dietary magnesium, which is associated with many aspects of cardiovascular health (23, 24). Magnesium may play a protective role in vascular calcification. Higher magnesium intake reduces the risk of stroke, non-fatal myocardial infarction, sudden cardiac death and fatal coronary artery disease (25–27). Magnesium is an antagonist of calcium, reversing plaque formation and calcification by inhibiting hydroxyapatite and crystal precipitation (28, 29). Previous studies have reported a negative correlation between serum magnesium levels and calcification in the vascular bed, such as carotid intima-media thickness and pulse wave velocity (30, 31). Results from a cross-sectional study showed a 22% reduction in the risk of coronary calcification for every 50 mg increase in dietary magnesium intake (*P* < 0.001). Those with the highest intake were 58% less likely to have any coronary calcification compared to those with the lowest intake of magnesium (32). Therefore, increased dietary magnesium intake under recommended upper limit (420 mg/day) (33) may reduce the risk of penile artery calcification and thus reduce the prevalence of ED.

People can supplement their diets with dried fruits, meats, milk, cereals, and eggs for zinc need (34). Zinc is a component of many metalloenzymes and involved in the synthesis of angiotensin converting enzyme, copper/zinc superoxide dismutase (35, 36). Therefore, zinc deficiency may lead to oxidative stress and inflammation, which can result in cardiovascular events. In addition, some studies have reported that zinc may help prevent atherosclerosis and endothelial damage (35). Zinc deficiency releases proatherogenic factors that lead to increased carotid intima-media thickness (37). Zinc deficiency may contribute to erectile dysfunction through distal small arterial hypertension (penile arteries), oxidative stress, and damage to the vascular endothelium. Dietary zinc intake is recommended to be no more than 40 mg/day (38), and the results of this study suggest that increasing dietary zinc intake within this recommended range may be beneficial in reducing the risk of ED.

Nuts, legumes, and dark chocolate are copper-rich foods (34). Copper is essential for enzyme function, with its dual role as a pro-oxidant and an antioxidant (39). Copper is a catalytic cofactor for enzymes involved in the formation of enzymes such as copper/zinc superoxide dismutase (SOD), copper cyanobactin (CPO) and lysyl oxidase. Both copper deficiency and overload play a key role in the formation of atherosclerosis (40). Copper deficiency decreases SOD activity, which promotes the formation of hydroxyl radicals and leads to atherosclerosis (40). In addition, copper deficiency affects cholesterol metabolism. Its deficiency decreases dehydroepiandrosterone synthesis, leading to arterial oxidative damage and thrombosis (41). Copper overload allows it to exert pro-oxidant properties that increase atherosclerosis formation (42). Therefore, copper concentrations should be maintained in a reasonable range. Increasing dietary copper intake within reasonable limits may reduce the risk of oxidative damage and thrombosis in penile arteries. To avoid causing organ damage, dietary copper should be less than 10 mg/day (38).

Fish and seafood are rich in selenium and are a good choice for dietary selenium supplementation (34). Selenium is also an important trace mineral, a component of selenoprotein, which has significant antioxidant properties (43). Selenium can reduce atherosclerosis, stroke and other cardiovascular events by inhibiting the toxicity of heavy metals (mercury, silver, arsenic, and cadmium), lipid peroxidation, and increasing plasma glutathione and nitric oxide concentrations (44, 45). Increased dietary selenium intake may reduce ED prevalence within the recommended range of 400 mcg/day.

However, there are some limitations in this study. First: dietary information related to erectile dysfunction was included only in the 2001 to 2004 survey, which may differ from the current population. Second: The respondent population was US residents. Due to the difference in ethnic composition, the results may not be applicable to men from Asian countries. Third: Self-assessed erectile dysfunction is a weak point in this study due to the lack of medical validation.

In conclusion, trace metals are involved in preventing vascular damage, atherosclerosis and thus protecting relatively small diameter penile arteries through multiple pathways. Increasing dietary metal intake up to the recommended upper limit is beneficial in reducing the prevalence of erectile dysfunction.

## Data availability statement

The original contributions presented in this study are included in the article/Supplementary material, further inquiries can be directed to the corresponding authors.

## Author contributions

R-JL designed the study and conducted the data extraction and analysis. R-JL, W-PM, Z-PX, S-YL, J-JY, and BX wrote the manuscript. R-JL, W-PM, Z-PX, CS, BX, and MC reviewed and revised the manuscript. All authors contributed to the article and approved the submitted version.

## Footnotes

1
https://www.cdc.gov/nchs/nhanes/index.htm
