# Supplementary material for: Dietary metal intake and the prevalence of erectile dysfunction in US men: Results from National Health and Nutrition Examination Survey 2001–2004
Source: Front Nutr. 2022 Nov 3;9:974443. doi: 10.3389/fnut.2022.974443 (PMC9668876; doi:10.3389/fnut.2022.974443)
Supplement: Supplementary file 3 [file Data_Sheet_1.docx]

Table 1 Baseline characteristics of NHANES participants between 2001 and 2004 before PSM.

| **Characteristic** | **Non-ED** | **ED** | **p** |
| --- | --- | --- | --- |
| Total patients | 2649 | 1096 |  |
| Age (years), n (%) |  |  | < 0.001 |
| <40 years | 1130 (30.2%) | 73 (1.9%) |  |
| ≥40 years | 1519 (40.6%) | 1023 (27.3%) |  |
| Race, n (%) |  |  | < 0.001 |
| Mexican American | 512 (13.7%) | 202 (5.4%) |  |
| Other Hispanic | 85 (2.3%) | 38 (1%) |  |
| Non-Hispanic White | 1428 (38.1%) | 674 (18%) |  |
| Non-Hispanic Black | 531 (14.2%) | 160 (4.3%) |  |
| Other Race - Including Multi-Racial | 93 (2.5%) | 22 (0.6%) |  |
| Education level, n (%) |  |  | < 0.001 |
| Less Than High School | 592 (15.8%) | 431 (11.5%) |  |
| High School Diploma | 692 (18.5%) | 220 (5.9%) |  |
| More Than High School | 1365 (36.4%) | 445 (11.9%) |  |
| Marital status, n (%) |  |  | < 0.001 |
| Married | 1580 (42.2%) | 784 (20.9%) |  |
| Unmarried | 1069 (28.5%) | 312 (8.3%) |  |
| Annual Household Income, n (%) |  |  | < 0.001 |
| Over $20,000 | 2190 (58.5%) | 788 (21%) |  |
| Under $20,000 | 459 (12.3%) | 308 (8.2%) |  |
| BMI (kg/m^2^), n (%) |  |  | 0.010 |
| <25.0 | 805 (21.5%) | 286 (7.6%) |  |
| ≥25.0 | 1844 (49.2%) | 810 (21.6%) |  |
| Hypertension, n (%) |  |  | < 0.001 |
| Yes | 631 (16.8%) | 584 (15.6%) |  |
| No | 2018 (53.9%) | 512 (13.7%) |  |
| Current health status, n (%) |  |  | < 0.001 |
| Excellent, very good, good | 2264 (60.5%) | 733 (19.6%) |  |
| Fair or poor | 385 (10.3%) | 363 (9.7%) |  |
| Diabetes, n (%) |  |  | < 0.001 |
| Yes | 134 (3.6%) | 256 (6.8%) |  |
| No | 2485 (66.4%) | 822 (21.9%) |  |
| Borderline | 30 (0.8%) | 18 (0.5%) |  |
| Magnesium (mg), median (IQR) | 301 (217, 407) | 256.5 (185.75, 335) | < 0.001 |
| Zinc (mg), median (IQR) | 12.76 (8.49, 18.04) | 10.1 (6.93, 14.64) | < 0.001 |
| Copper (mg), median (IQR) | 1.34 (0.94, 1.83) | 1.1 (0.82, 1.5) | < 0.001 |
| Selenium (mcg), median (IQR) | 117.3 (85.1, 160.7) | 97.4 (71.57, 130.83) | < 0.001 |

For categorical variables, P values were analyzed by chi-square tests. For continuous variables, P values

were analyzed by Wilcoxon rank sum test. BMI, body mass index; PSM, propensity score matching; IQR, interquartile range.

Table 2. Subgroup analysis of dietary metal intake and erectile dysfunction in NHANES 2001–2004 before PSM.

| **Characteristic** |  | **OR (95% CI)** |  | **P value** |
| --- | --- | --- | --- | --- |
| **Magnesium** | **100mg / day** | **200mg / day** | **300mg / day** |  |
| Overall | 1.375 (1.069-1.769) | 1.285 (1.185-1.393) | 0.933 (0.909-0.957) | <.0001 |
| <40 years | 1.488 (0.677-3.270) | 1.311 (0.982-1.750) | 1.028 (0.990-1.068) | 0.1747 |
| ≥40 years | 1.356 (1.013-1.816) | 1.233 (1.133-1.343) | 0.923 (0.887-0.961) | <.0001 |
| BMI<25.0 kg/m^2^ | 1.528 (0.998-2.340) | 1.426 (1.209-1.681) | 0.959 (0.938-0.981) | <.0001 |
| BMI≥25.0 kg/m^2^ | 1.356 (0.991-1.856) | 1.243 (1.133-1.363) | 0.938 (0.907-0.970) | <.0001 |
| Married | 1.525 (1.110-2.096) | 1.331 (1.202-1.475) | 0.952 (0.931-0.974) | <.0001 |
| Unmarried | 1.333 (0.891-1.994) | 1.271 (1.121-1.441) | 0.906 (0.845-0.960) | <.0001 |
| **Zinc** | **10mg / day** | **20mg / day** | **30mg / day** |  |
| Overall | 1.152 (1.115-1.189) | 0.564 (0.479-0.664) | 0.531 (0.438-0.644) | <.0001 |
| <40 years | 1.227 (1.020-1.475) | 0.909 (0.595-1.387) | 1.045 (0.597-1.825) | 0.0475 |
| ≥40 years | 1.075 (1.049-1.102) | 0.595 (0.491-0.719) | 0.563 (0.452-0.701) | <.0001 |
| BMI<25.0 kg/m^2^ | 1.375 (1.265-1.493) | 0.334 (0.242-0.462) | 0.329 (0.223-0.484) | <.0001 |
| BMI≥25.0 kg/m^2^ | 1.116 (1.072-1.162) | 0.690 (0.571-0.834) | 0.642 (0.521-0.804) | <.0001 |
| Married | 1.159 (1.114-1.206) | 0.558 (0.458-0.681) | 0.536 (0.423-0.677) | <.0001 |
| Unmarried | 1.161 (0.084-1.242) | 0.613 (0.459-0.817) | 0.548 (0.384-0.781) | <.0001 |
| **Copper** | **1mg / day** | **2mg / day** | **3mg / day** |  |
| Overall | 1.250 (1.197-1.305) | 0.534 (0.458-0.622) | 0.482 (0.402-0.579) | <.0001 |
| <40 years | 1.201 (0.986-1.464) | 0.864 (0.558-1.340) | 0.813 (0.445-1.485) | 0.3127 |
| ≥40 years | 1.175 (1.128-1.225) | 0.563 (0.470-0.674) | 0.539 (0.440-0.660) | <.0001 |
| BMI<25.0 kg/m^2^ | 1.342 (1.225-1.471) | 0.451 (0.333-0.610) | 0.338 (0.228-0.502) | <.0001 |
| BMI≥25.0 kg/m^2^ | 1.244 (1.176-1.316) | 0.566 (0.472-0.680) | 0.553 (0.447-0.684) | <.0001 |
| Married | 1.296 (1.216-1.381) | 0.569 (0.472-0.686) | 0.534 (0.430-0.664) | <.0001 |
| Unmarried | 1.241 (1.162-1.324) | 0.455 (0.338-0.613) | 0.403 (0.285-0.572) | <.0001 |
| **Selenium** | **100mcg / day** | **200mcg / day** | **300mcg / day** |  |
| Overall | 1.121 (1.086-1.158) | 0.510 (0.430-0.606) | 0.431 (0.317-0.585) | <.0001 |
| <40 years | 1.093 (0.910-1.314) | 1.050 (0.634-.1740) | 1.049 (0.487-2.257) | 0.1210 |
| ≥40 years | 1.056 (1.034-1.077) | 0.548 (0.452-0.664) | 0.478 (0.342-0.668) | <.0001 |
| BMI<25.0 kg/m^2^ | 1.147 (1.086-1.211) | 0.340 (0.240-0.481) | 0.258 (0.129-0.513) | <.0001 |
| BMI≥25.0 kg/m^2^ | 1.095 (1.057-1.134) | 0.591 (0.485-0.720) | 0.514 (0.362-0.731) | <.0001 |
| Married | 1.118 (1.075-1.163) | 0.553 (0.451-0.678) | 0.515 (0.362-0.733) | <.0001 |
| Unmarried | 1.135 (1.072-1.200) | 0.452 (0.329-0.620) | 0.339 (0.189-0.611) | <.0001 |

CI, confidence interval; OR, odds ratio; PSM, propensity score matching.

Table 3. Multivariate logistic regression analysis of the relationship between dietary metal intake and erectile dysfunction in NHANES 2001–2004 before PSM.

| **Metals** | **Magnesium** | | **Zinc** | | **Copper** | | **Selenium** | |
| --- | --- | --- | --- | --- | --- | --- | --- | --- |
|  | **OR^*^ (95% CI)** | **P** | **OR^*^ (95% CI)** | **P** | **OR^*^ (95% CI)** | **P** | **OR^*^ (95% CI)** | **P** |
| Q1 | Ref |  | Ref |  | Ref |  | Ref |  |
| Q2 | 0.930 (0.746-1.161) | 0.522 | 0.901 (0.725-1.121) | 0.350 | 0.977 (0.784-1.217) | 0.833 | 0.942 (0.785-1.170) | 0.587 |
| Q3 | 0.743 (0.592-0.932) | 0.010 | 0.743 (0.593-0.931) | 0.010 | 0.781 (0.622-0.982) | 0.034 | 0.726 (0.582-0.907) | 0.005 |
| Q4 | 0.533 (0.418-0.679) | <0.001 | 0.557 (0.439-0.709) | <0.001 | 0.535 (0.419-0.683) | <0.001 | 0.554 (0.436-0.705) | <0.001 |
| P for trend | <0.001 |  | <0.001 |  | <0.001 |  | <0.001 |  |

CI, confidence interval; OR, odds ratio; PSM, propensity score matching; ^*^, adjust for age, race, education level, marital status, annual

household income, BMI, hypertension, diabetes, current health status.

Table 4 Baseline characteristics of NHANES participants between 2001 and 2004 after PSM.

| **Characteristic** | **Non-ED** | **ED** | **p** |
| --- | --- | --- | --- |
| Total patients | 896 | 896 |  |
| Age (years), n (%) |  |  | 0.864 |
| <40 years | 76 (4.2%) | 73 (4.1%) |  |
| ≥40 years | 820 (45.8%) | 823 (45.9%) |  |
| Race, n (%) |  |  | 0.005 |
| Mexican American | 160 (8.9%) | 155 (8.6%) |  |
| Other Hispanic | 26 (1.5%) | 32 (1.8%) |  |
| Non-Hispanic White | 529 (29.5%) | 584 (32.6%) |  |
| Non-Hispanic Black | 161 (9%) | 106 (5.9%) |  |
| Other Race - Including Multi-Racial | 20 (1.1%) | 19 (1.1%) |  |
| Education level, n (%) |  |  | 0.171 |
| Less Than High School | 302 (16.9%) | 315 (17.6%) |  |
| High School Diploma | 215 (12%) | 182 (10.2%) |  |
| More Than High School | 379 (21.1%) | 399 (22.3%) |  |
| Marital status, n (%) |  |  | 1.000 |
| Married | 625 (34.9%) | 624 (34.8%) |  |
| Unmarried | 271 (15.1%) | 272 (15.2%) |  |
| Annual Household Income, n (%) |  |  | 0.482 |
| Over $20,000 | 659 (36.8%) | 673 (37.6%) |  |
| Under $20,000 | 237 (13.2%) | 223 (12.4%) |  |
| BMI (kg/m^2^), n (%) |  |  | 1.000 |
| <25.0 | 236 (13.2%) | 237 (13.2%) |  |
| ≥25.0 | 660 (36.8%) | 659 (36.8%) |  |
| Hypertension, n (%) |  |  | 0.297 |
| Yes | 428 (23.9%) | 405 (22.6%) |  |
| No | 468 (26.1%) | 491 (27.4%) |  |
| Current health status, n (%) |  |  | 0.418 |
| Excellent, very good, good | 673 (37.6%) | 657 (36.7%) |  |
| Fair or poor | 223 (12.4%) | 239 (13.3%) |  |
| Diabetes, n (%) |  |  | 0.017 |
| Yes | 115 (6.4%) | 140 (7.8%) |  |
| No | 774 (43.2%) | 738 (41.2%) |  |
| Borderline | 7 (0.4%) | 18 (1%) |  |
| Magnesium (mg), median (IQR) | 280 (202, 393.25) | 260 (191, 338.25) | < 0.001 |
| Zinc (mg), median (IQR) | 11.41 (7.55, 16.5) | 10.34 (7.06, 14.74) | 0.002 |
| Copper (mg), median (IQR) | 1.25 (0.88, 1.72) | 1.11 (0.84, 1.5) | < 0.001 |
| Selenium (mcg), median (IQR) | 108 (78.15, 147.25) | 98.4 (71.68, 130.75) | < 0.001 |

For categorical variables, P values were analyzed by chi-square tests. For continuous variables, P values

were analyzed by Wilcoxon rank sum test. BMI, body mass index; PSM, propensity score matching. IQR, interquartile range.

Table 5. Subgroup analysis of dietary metal intake and erectile dysfunction in NHANES 2001–2004 after PSM.

| **Characteristic** |  | **OR (95% CI)** |  | **P value** |
| --- | --- | --- | --- | --- |
| **Magnesium** | **100mg / day** | **200mg / day** | **300mg / day** |  |
| Overall | 1.012 (1.005-1.019) | 0.626 (0.503-0.780) | 0.572 (0.402-0.815) | <.0001 |
| <40 years | 1.311 (0.494-3.477) | 1.244 (0.897-1.726) | 0.911 (0.780-1.064) | 0.547 |
| ≥40 years | 0.811 (0.571-1.153) | 1.077 (0.980-1.182) | 0.906 (0.845-0.970) | <.0001 |
| BMI<25.0 kg/m^2^ | 0.728 (0.402-1.319) | 1.093 (0.919-1.301) | 0.867 (0.755-0.996) | 0.0011 |
| BMI≥25.0 kg/m^2^ | 0.887 (0.583-1.347) | 1.100 (0.981-1.234) | 0.913 (0.848-0.983) | 0.0096 |
| Married | 0.755 (0.488-1.167) | 1.052 (0.933-1.186) | 0.929 (0.870-0.993) | 0.0020 |
| Unmarried | 0.976 (0.563-1.692) | 1.151 (1.010-1.313) | 0.854 (0.754-0.968) | 0.0059 |
| **Zinc** | **10mg / day** | **20mg / day** | **30mg / day** |  |
| Overall | 1.048 (1.019-1.077) | 0.677 (0.545-0.841) | 0.665 (0.520-0.850) | 0.0039 |
| <40 years | 0.997 (0.803-1.239) | 1.098 (0.617-1.954) | 1.185 (0.602-2.330) | 0.902 |
| ≥40 years | 1.044 (1.019-1.071) | 0.634 (0.503-0.799) | 0.606 (0.464-0.791) | 0.0010 |
| BMI<25.0 kg/m^2^ | 1.035 (1.016-1.054) | 0.415 (0.263-0.655) | 0.419 (0.255-0.689) | 0.0021 |
| BMI≥25.0 kg/m^2^ | 1.031 (0.996-1.068) | 0.779 (0.606-1.001) | 0.755 (0.566-1.007) | 0.231 |
| Married | 1.044 (1.012-1.078) | 0.672 (0.516-0.874) | 0.662 (0.493-0.888) | 0.0295 |
| Unmarried | 1.036 (0.996-1.077) | 0.669 (0.451-0.992) | 0.645 (0.397-1.047) | 0.1746 |
| **Copper** | **1mg / day** | **2mg / day** | **3mg / day** |  |
| Overall | 1.119 (1.070-1.170) | 0.559 (0.451-0.692) | 0.544 (0.430-0.689) | <.0001 |
| <40 years | 1.130 (0.880-1.451) | 0.842 (0.438-1.618) | 0.945 (0.442-2.022) | 0.786 |
| ≥40 years | 1.123 (1.073-1.175) | 0.536 (0.429-0.671) | 0.523 (0.410-0.668) | <.0001 |
| BMI<25.0 kg/m^2^ | 1.081 (1.017-1.149) | 0.500 (0.322-0.774) | 0.422 (0.252-0.704) | 0.0095 |
| BMI≥25.0 kg/m^2^ | 1.134 (1.069-1.204) | 0.557 (0.432-0.719) | 0.656 (0.468-0.918) | 0.0001 |
| Married | 1.149 (1.070-1.235) | 0.543 (0.419-0.703) | 0.586 (0.429-0.795) | 0.0001 |
| Unmarried | 1.100 (0.036-1.168) | 0.549 (0.373-0.802) | 0.529 (0.349-0.802) | 0.0180 |
| **Selenium** | **100mcg / day** | **200mcg / day** | **300mcg / day** |  |
| Overall | 1.012 (1.005-1.019) | 0.626 (0.503-0.780) | 0.572 (0.402-0.815) | 0.0001 |
| <40 years | 0.975 (0.774-1.227) | 1.230 (0.643-2.350) | 1.350 (0.590-3.093) | 0.885 |
| ≥40 years | 1.016 (1.007-1.025) | 0.578 (0.459-0.726) | 0.512 (0.348-0.752) | <.0001 |
| BMI<25.0 kg/m^2^ | 1.003 (1.000-1.005) | 0.430 (0.280-0.662) | 0.378 (0.215-0.664) | 0.0019 |
| BMI≥25.0 kg/m^2^ | 1.027 (1.004-1.051) | 0.782 (0.566-0.936) | 0.703 (0.459-1.077) | 0.0102 |
| Married | 1.025 (1.008-1.041) | 0.611 (0.471-0.794) | 0.586 (0.384-0.893) | 0.0031 |
| Unmarried | 0.998 (0.996-1.000) | 0.659 (0.446-0.975) | 0.570 (0.325-1.001) | 0.025 |

CI, confidence interval; OR, odds ratio; PSM, propensity score matching.

Table 6. Multivariate logistic regression analysis of the relationship between dietary metal intake and erectile dysfunction in NHANES 2001–2004 after PSM.

| **Metals** | **Magnesium** | | **Zinc** | | **Copper** | | **Selenium** | |
| --- | --- | --- | --- | --- | --- | --- | --- | --- |
|  | **OR^*^ (95% CI)** | **P** | **OR^*^ (95% CI)** | **P** | **OR^*^ (95% CI)** | **P** | **OR^*^ (95% CI)** | **P** |
| Q1 | Ref |  | Ref |  | Ref |  | Ref |  |
| Q2 | 0.949 (0.726-1.242) | 0.704 | 0.908 (0.694-1.190) | 0.485 | 1.175 (0.897-1.538) | 0.241 | 0.985 (0.754-1.286) | 0.909 |
| Q3 | 0.904 (0.689-1.186) | 0.465 | 0.817 (0.623-1.069) | 0.141 | 0.822 (0.627-1.079) | 0.157 | 0.841 (0.644-1.099) | 0.204 |
| Q4 | 0.551 (0.419-0.724) | <0.001 | 0.645 (0.491-0.847) | 0.002 | 0.557 (0.423-0.733) | <0.001 | 0.538 (0.410-0.706) | <0.001 |
| P for trend | <0.001 |  | 0.011 |  | <0.001 |  | <0.001 |  |

CI, confidence interval; OR, odds ratio; PSM, propensity score matching; ^*^, adjust for age, race, education level, marital status, annual

household income, BMI, hypertension, diabetes, current health status.

Table 7. Food sources of trace metals and recommended upper limit.

| **Metals** | **Food sources of trace metals** | **Dietary Reference upper limit** |
| --- | --- | --- |
| **Magnesium** | Grains, green leafy vegetables, almonds, coffee and dark chocolate | 420 mg/day |
| **Zinc** | Dry fruits (especially pine nuts, peanuts, nuts and almonds), all kind of meat, milk, cereals and eggs | 40 mg/day |
| **Copper** | Dry fruits, pine nuts, hazelnuts, soy beans, black-eyed beans/peas and lentils, dark chocolate | 10mg/day |
| **Selenium** | Fish, seafood, eggs, offal and red meat | 400 mcg/day |
