# Supplementary material for: Dietary metal intake and the prevalence of erectile dysfunction in US men: Results from National Health and Nutrition Examination Survey 2001–2004
Source: Front Nutr. 2022 Nov 3;9:974443. doi: 10.3389/fnut.2022.974443 (PMC9668876; doi:10.3389/fnut.2022.974443)
Supplement: Supplementary file 4 [file Data_Sheet_2.docx]

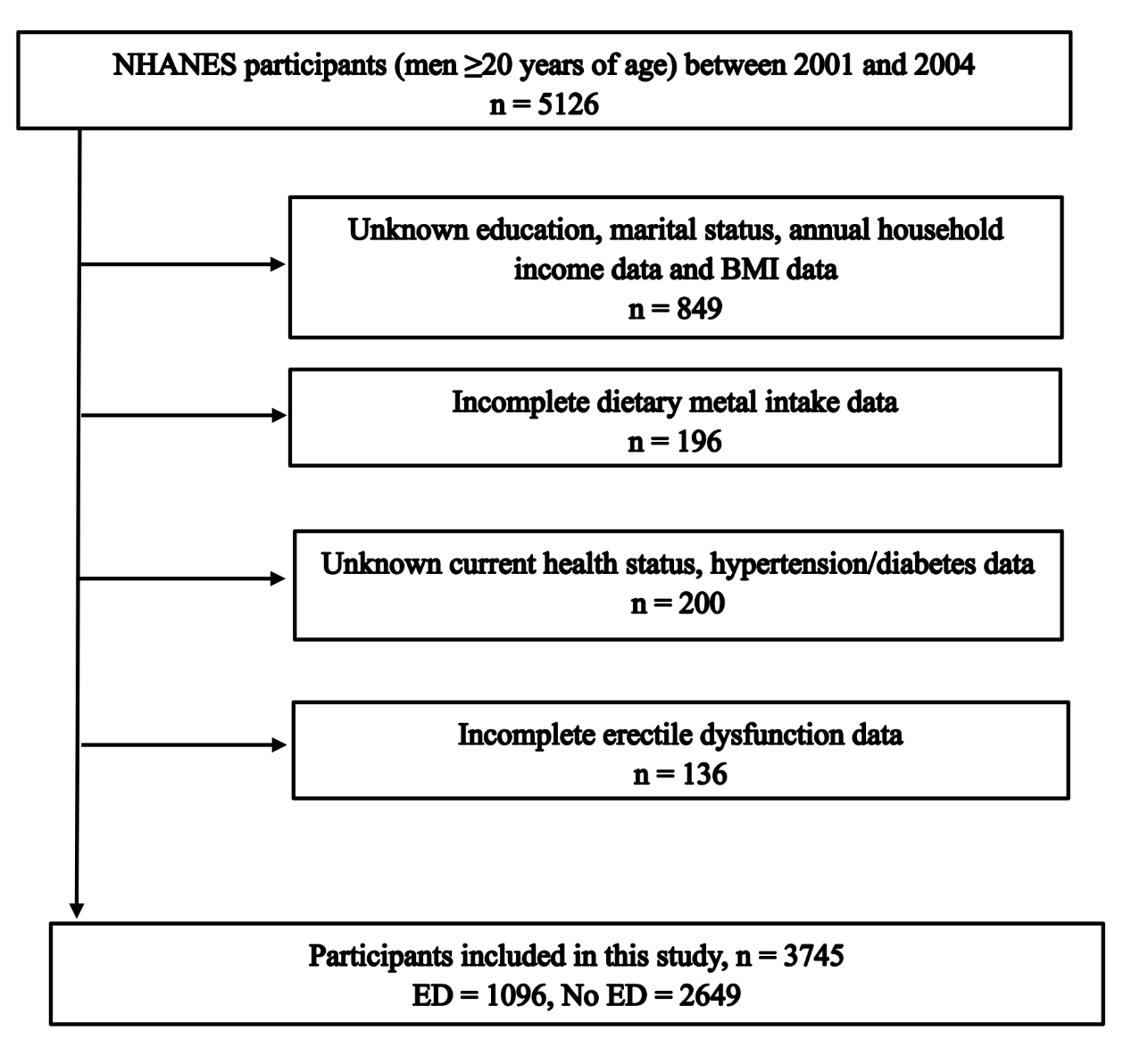


**Figure 1.** Flow chart of the study population identification.


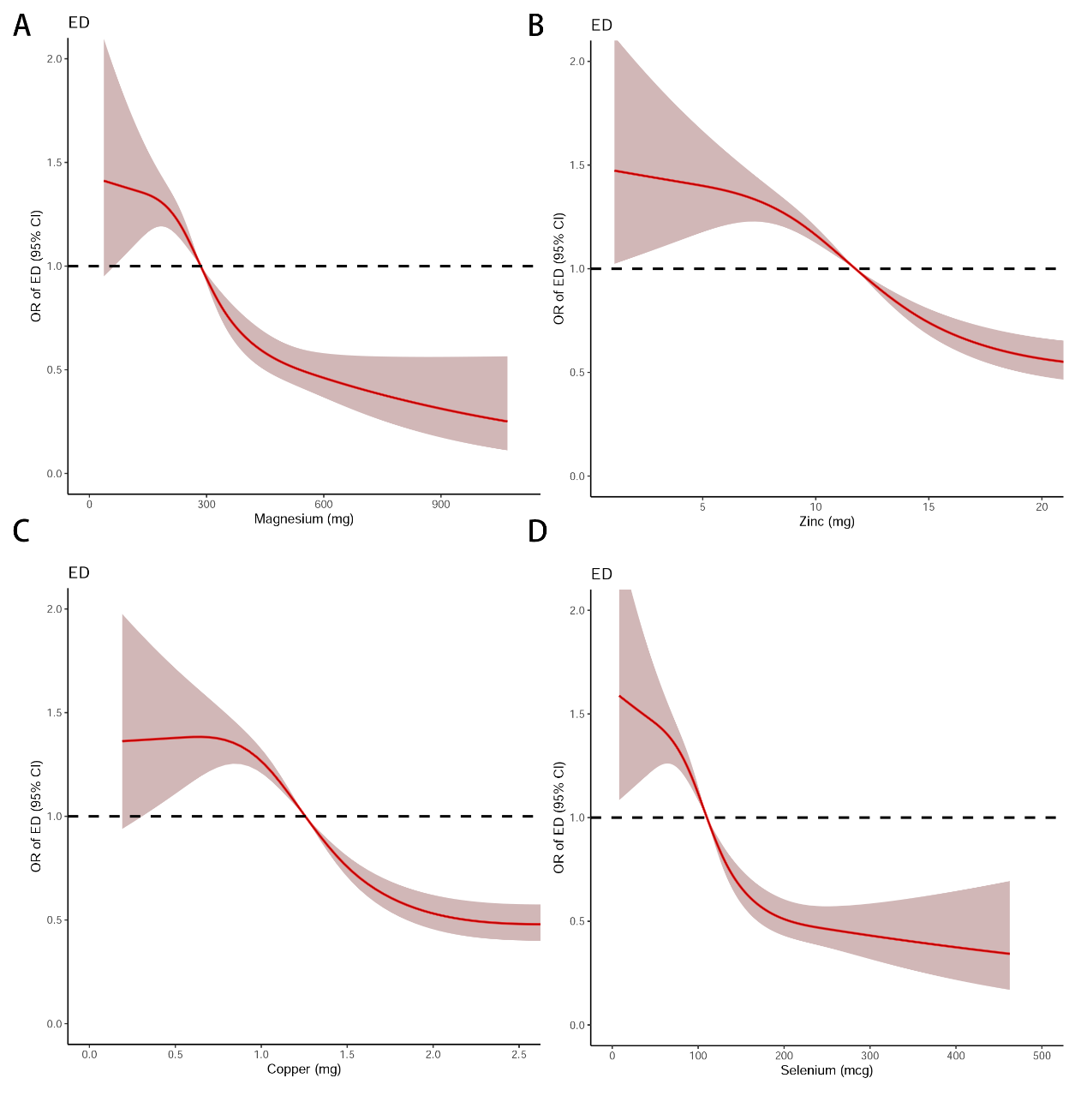


**Figure 2.** The dose-response analysis of dietary metal intake and erectile dysfunction before propensity score matching. (A) Magnesium; (B) Zinc; (C) Copper; (D) Selenium.

**
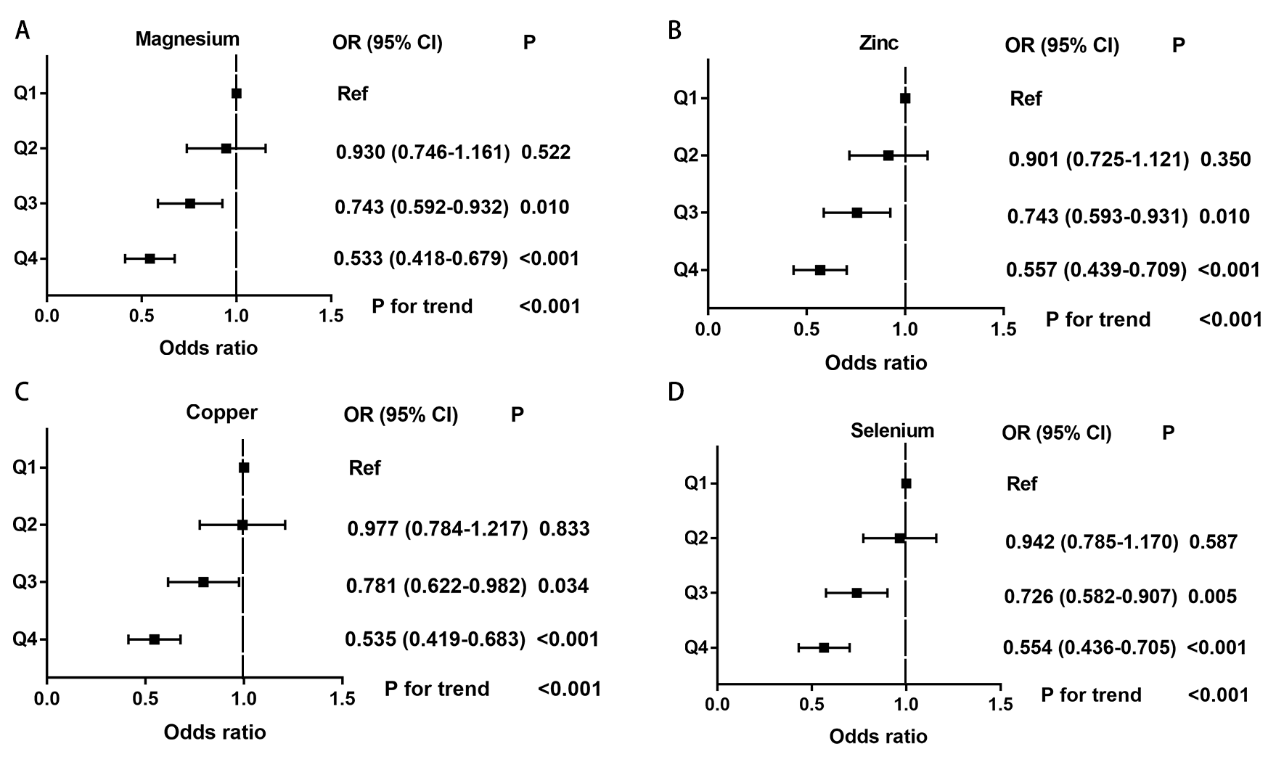
**

**Figure 3.** The adjusted odds ratios (95% CI) of dietary metal intake and erectile dysfunction before propensity score matching. (A) Magnesium; (B) Zinc; (C) Copper; (D) Selenium.


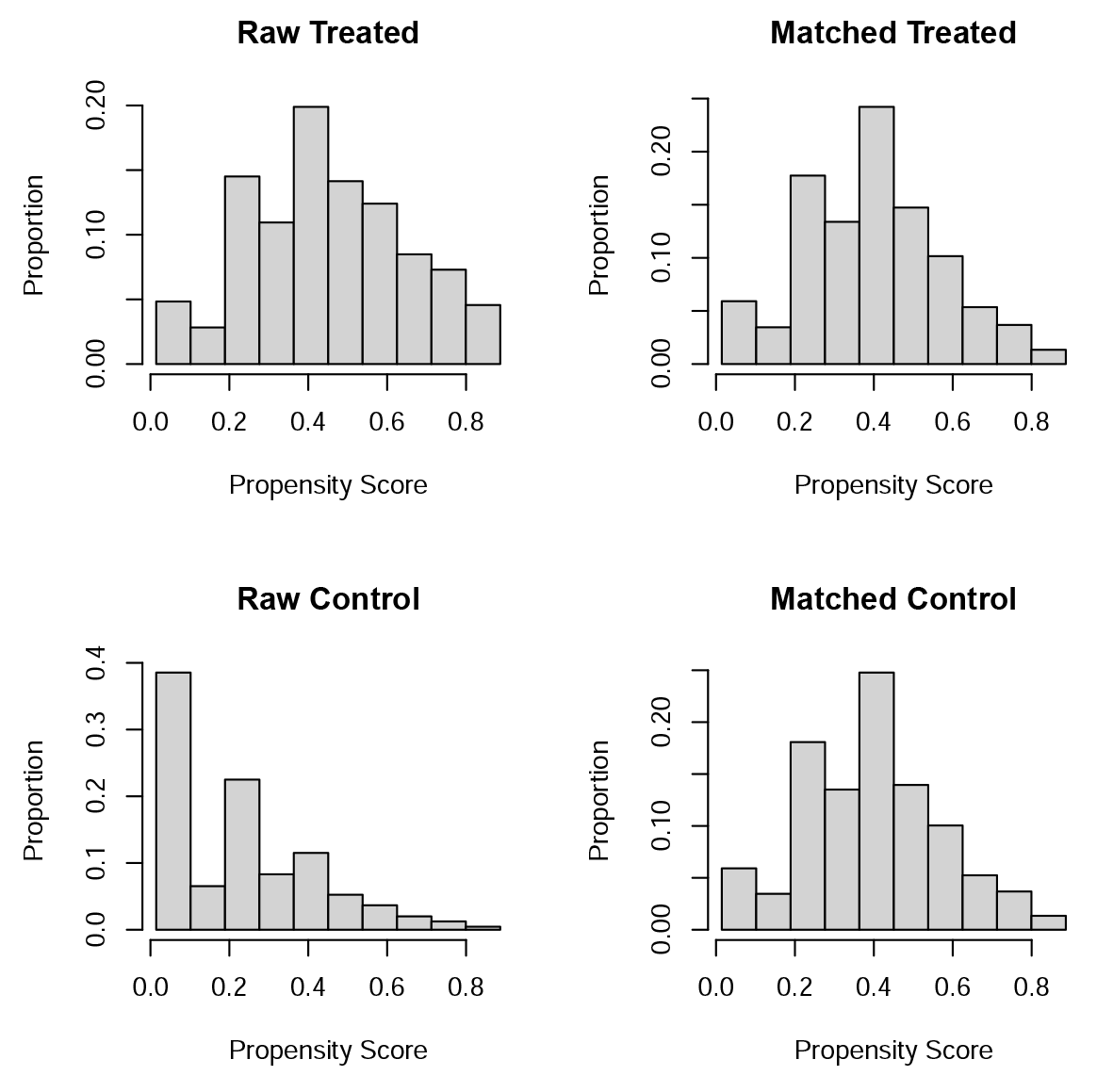


**Figure 4.** Propensity score matching analysis for treated and control population.


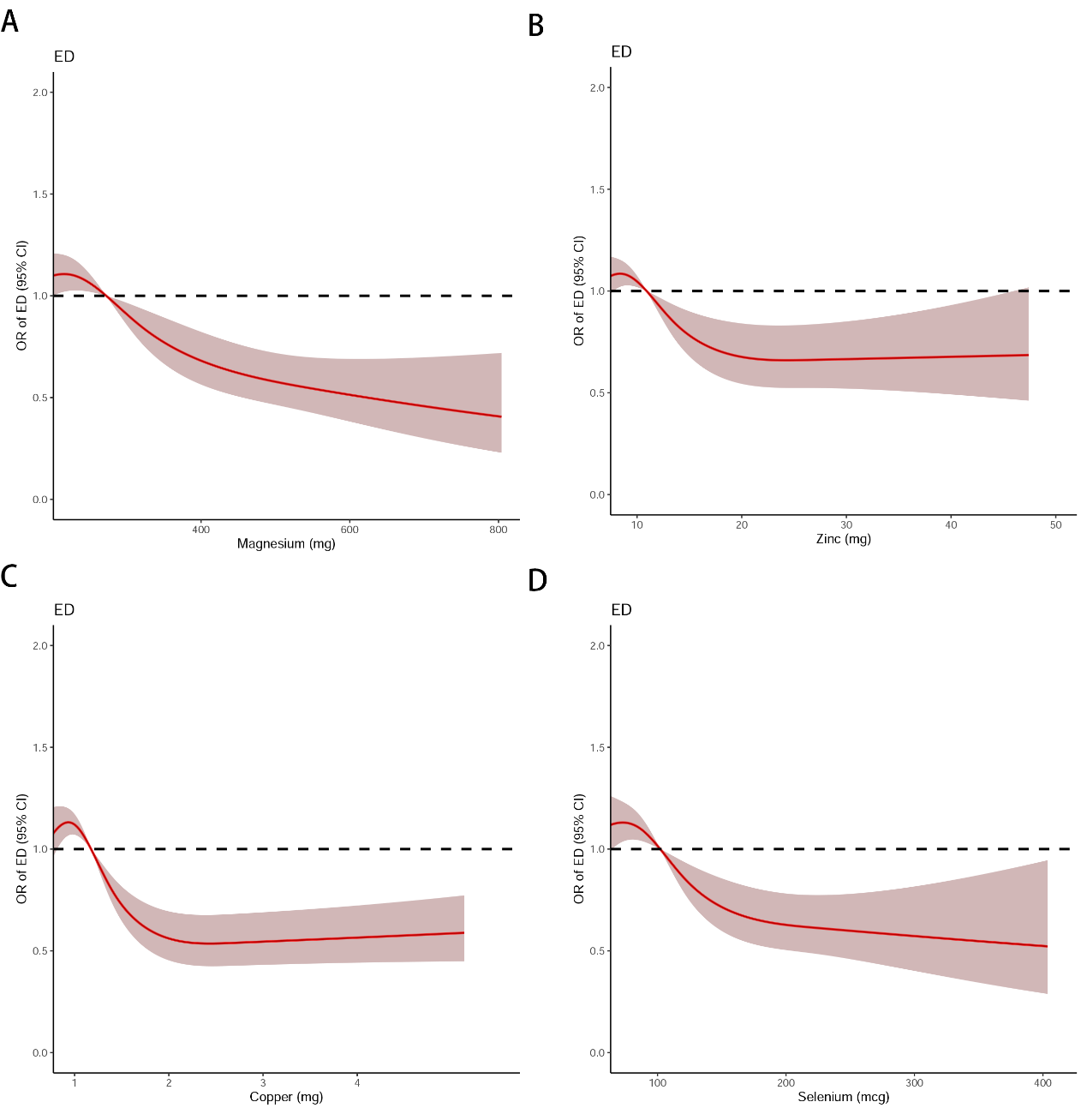


**Figure 5.** The dose-response analysis of dietary metal intake and erectile dysfunction after propensity score matching. (A) Magnesium; (B) Zinc; (C) Copper; (D) Selenium.


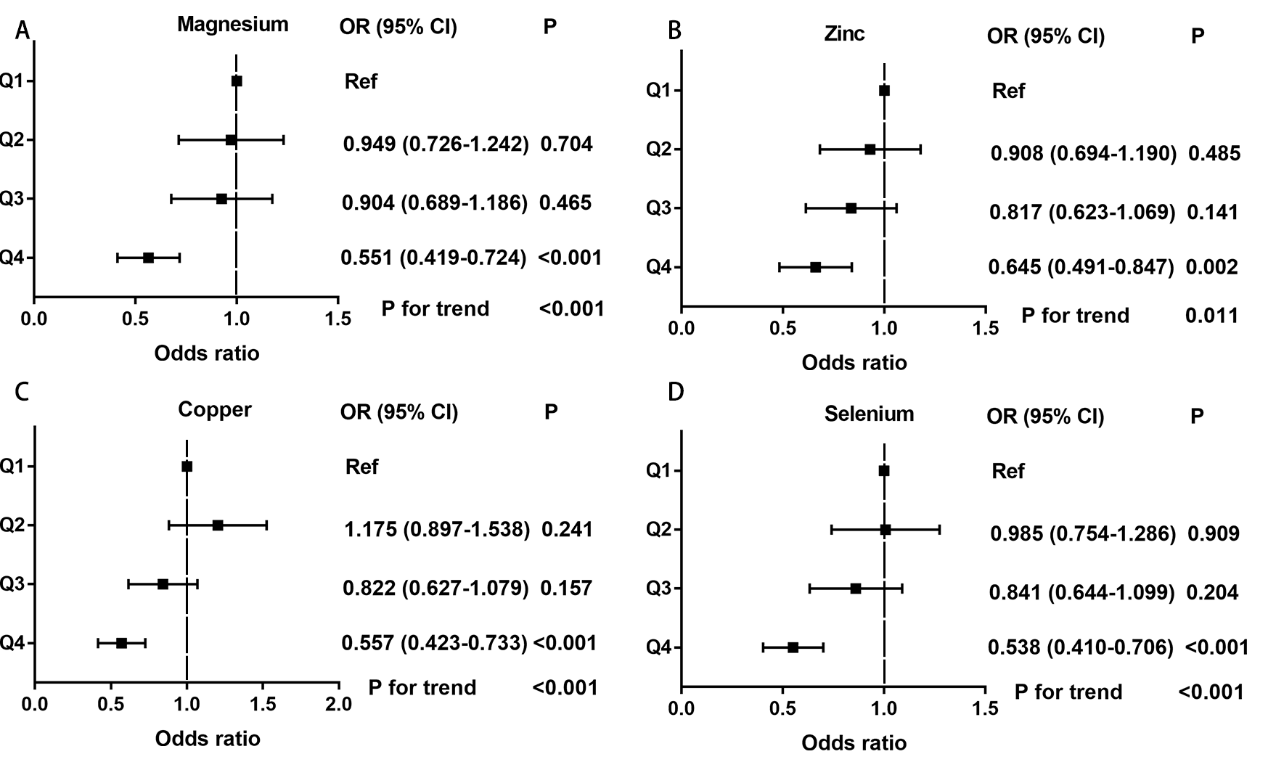


**Figure 6.** The adjusted odds ratios (95% CI) of dietary metal intake and erectile dysfunction after propensity score matching. (A) Magnesium; (B) Zinc; (C) Copper; (D) Selenium.
